# Insecticidal decay effects of long-lasting insecticide nets and indoor residual spraying on *Anopheles gambiae* and *Anopheles arabiensis* in Western Kenya

**DOI:** 10.1186/s13071-015-1194-6

**Published:** 2015-11-14

**Authors:** Christine L. Wanjala, Guofa Zhou, Jernard Mbugi, Jemimah Simbauni, Yaw A. Afrane, Ednah Ototo, Maxwell Gesuge, Harrysone Atieli, Andrew K. Githeko, Guiyun Yan

**Affiliations:** Centre for Global Health Research, Kenya Medical Research Institute, Kisumu, Kenya; Departments of Zoological Sciences, Kenyatta University, Nairobi, Kenya; Department of Medical Laboratory Sciences, Masinde Muliro University of Science and Technology, Kakamega, Kenya; Program in Public Health, University of California, Irvine, CA 92697 USA

**Keywords:** Malaria, Insecticidal decay, Insecticide resistance, Indoor residual spraying, Long-lasting insecticide nets

## Abstract

**Background:**

Indoor residual spraying (IRS) and long-lasting insecticidal nets (LLINs) are the first-line tools for malaria prevention and control in Africa. Vector resistance to insecticides has been extensively studied, however the insecticidal effects of the nets and sprayed walls on pyrethroid resistant mosquitoes has not been studied thoroughly. We evaluated the bioefficacy of LLINs of different ages and lambda-cyhalothrin (ICON 10cs) on the sprayed mud walls for a period of time on malaria vector survivorship.

**Methods:**

WHO tube bioassay was performed using diagnostic doses of lambda-cyhalothrin (0.05 %), permethrin (0.75 %) and deltamethrin (0.05 %). Cone bioassays were conducted on netting materials from 0 to 3 years old long-lasting insecticide-impregnated nets. Wall bioassays were performed monthly on mud slabs sprayed with lambdacyhalothrin over a period of seven months. All bioassays used *An. gambiae* mosquitoes collected from the field and the laboratory susceptible reference Kisumu strain. Concentration of the insecticides on the netting materials was examined using the gas chromatography method. Mosquitoes were identified to species level using PCR and genotyped for the *kdr* gene mutation frequencies.

**Results:**

WHO bioassays results showed that populations from five sites were highly resistant to the pyrethroids (mortalities ranged from 52.5 to 75.3 %), and two sites were moderately resistant to these insecticides (80.4 – 87.2 %). Homozygote *kdr* mutations of L1014S ranged from 73 to 88 % in *An. gambiae* s.s. dominant populations whereas L1014S mutation frequencies were relatively low (7–31 %) in *An. arabiensis* dominant populations. There was a significant decrease (*P* < 0.05) in mosquito mortality with time after the spray with both lambda-cyhalothrin (75 % mortality after six months) and with the age of LLINs (60 % mortality after 24 month). Field collected mosquitoes were able to survive exposure to both IRS and LLINs even with newly sprayed walls (86.6–93.5 % mortality) and new LLINs (77.5–85.0 % mortality), Wild mosquitoes collected from the field had significantly lower mortality rates to LLINs (59.6–85.0 %) than laboratory reared susceptible strain (100 %). Insecticide concentration decreased significantly from 0.14 μg/ml in the new nets to 0.077 μg/ml in nets older than 18 months (*P* < 0.05).

**Conclusion:**

This study confirms that insecticide decay and developing levels of resistance have a negative contribution to reduced efficacy of ITN and IRS in western Kenya. These factors contribute to decreased efficacy of pyrethroid insectides in ongoing malaria control programs. In order to mitigate against the impact of insecticide resistance and decay it is important to follow the WHO policy to provide the residents with new LLINs every three years of use while maintaining a high level of LLINs coverage and usage. There is also need for urgent development and deployment of non-pyrethroid based vector control tools.

## Background

Despite global efforts to malaria control, malaria remains a major public health problem particularly in Africa where more than 80 % of the cases are reported [1]. Long lasting insecticide nets (LLINs), indoor residual spraying (IRS) and case management using artemisinin-combination therapy are the key tools currently used for malaria control [1]. There are indications of variations in compliance and insecticide resistance in Kenya [2, 3], however the role of insecticide decay in LLINs and IRS is not well understood in the context of high insecticide resistance. The efficacy of LLIN and IRS for malaria control depends on the adherence to the specified insecticide application procedure, insecticide resistance in mosquito population and the persistence of the insecticides on the sprayed surface. Mosquito resistance to insecticide, which reduces the efficacy of LLINs, has been intensively studied across Africa, and resistance has been detected in all major African malaria vectors and resistance to multiple classes of insecticides is not uncommon [4–15]. However, insecticides may lose their effectiveness due to chemical degradation over time and bioavailability of the insecticide on the sprayed surface which may be affected by the porosity of the sprayed surface [16]. Reduced effectiveness of ITN/LLIN and IRS control programs can contribute to an upsurge of malaria incidence [17, 18].

The decay of insecticidal efficacy with time in IRS program has been previously studied. For example, re-spray was not needed after 6 to 12 months of DDT spray, but houses sprayed with lambda-cyhalothrin would need to be re-sprayed every 3–4 months to maintain acceptable efficacies [19]. Insecticidal efficacy decay is less clear in LLINs, particularly when mosquitoes are modestly resistant to pyrethroids. Three classes of LLINs are recommended by WHO: 1) permethrin-incorporated net - a LLIN made of high density polyethylene monofilament yarn blended with 2 % permethrin, 2) deltamethrin-coated net – a LLIN made of multifilament polyester netting treated with deltamethrin, and 3) alphacypermethrin-coated net – a LLIN made of multifilament polyester netting treated with alphacypermethrin [20]. Currently 11 brands of LLINs are recommended by WHO for public use: 1) DawaPlus 2.0 - Deltamethrin coated on polyester; 2) Duranet - Alpha-cypermethrin incorporated into polyethylene; 3) Interceptor - Alpha-cypermethrin coated on polyester; 4) LifeNet - Deltamethrin incorporated into polypropylene; 5) MAGNet - Alpha-cypermethrin incorporated into polyethylene; 6) Olyset Net - Permethrin incorporated into polyethylene; 7) Olyset Plus - Permethrin and PBO incorporated into polyethylene; 8)PermaNet 2.0 - Deltamethrin coated on polyester; 9) PermaNet 3.0 - Combination of deltamethrin coated on polyester with strengthened border (side panels), deltamethrin and PBO incorporated into polyethylene (roof); 10) Royal Sentry- Alpha-cypermethrin incorporated into polyethylene; and 11) Yorkool LN - Deltamethrin coated on polyester [20]. Duration of protective efficacy should be at least 3 years under recommended conditions of use. Indeed, a number of studies reported reduced efficacy of LLINs after several years of use [18, 21, 22]. Some nets have been shown to be less effective in inhibiting mosquito feeding [23], and wear and washing of nets may cause further insecticidal efficacy decay [24].

Intensive pyrethroid-based malaria vector control program in the past decade in Africa has led to rapid rise of pyrethroid resistance in malaria vectors across Africa. For example, pyrethroid resistance was reported in *An. gambiae* in 27 sub-Saharan African countries, in *An. arabiensis* in 14 sub-Saharan countries, and *An. funestus* in at least 4 countries [14]. In addition to being geographically widespread, knockdown resistance (*kdr*) mutations have reached to extremely high frequency levels for *An. gambiae* throughout Africa [13, 25–31]. The scale up of ITN and IRS programs will select for higher insecticide resistance [32]. Important questions related to the first-line malaria vector control tools include: how effective are old LLINs when mosquito vectors are modestly or highly resistant to the insecticide used in the intervention, and how often should a house be re-sprayed after one round of IRS?

The objectives of this study were to assess the impact of insecticidal decay effect in the context of modest to high insecticide resistance. Comprehensive information on bioefficacy and persistence of the insecticide on the LLINs and indoor residual sprayed walls in the context of vector insecticide resistance is important for making operational decisions in malaria control programmes, such as the frequency of net replacement and re-spray in IRS program under the current situation of modest to high pyrethroid resistance in the malaria vector populations.

## Methods

### Study sites

**Fig. 1:**
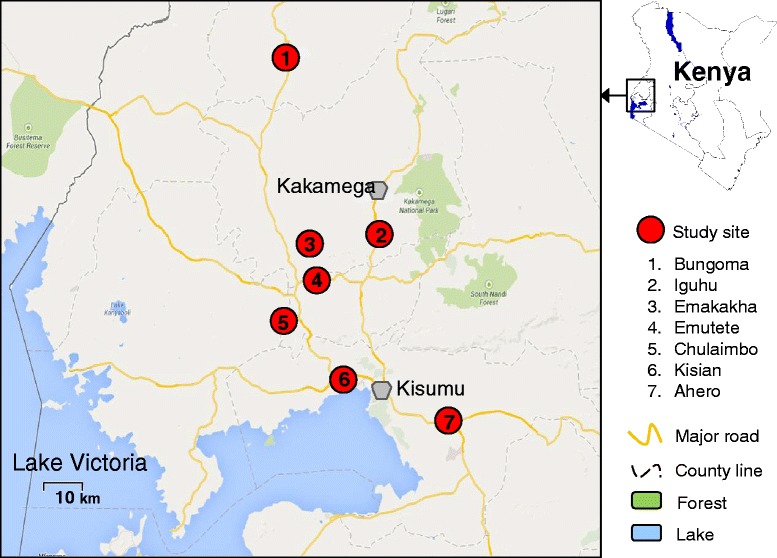
A map of the study sites in western Kenya

The study was conducted in seven villages in western Kenya: Iguhu, Emakakha, Chulaimbo, Emutete, Bungoma, Ahero and Kisian (Fig. 1). Bungoma, Emutete, Iguhu and Emakakha are in the highland-fringe malaria epidemic area whereas Chulaimbo, Ahero and Kisian are in the basin region of Lake Victoria (low land) where malaria is endemic. *An. gambiae* is the predominant species in Bungoma, Emutete, Iguhu, Emakakha, Kisian and Chulaimbo, whereas *An. arabiensis* is the predominant species in Ahero [33–35], however there was a significant malaria vector species composition change recently [36]. Bungoma and Emutete were previously used for other insecticide resistance studies [33, 37]. Maize is the primary agricultural crop for all sites except that Ahero has irrigation and rice is a main crop.

### Scientific and ethical clearance

This study was approved by the Ethical Review Board of the Kenya Medical Research Institute (KEMRI) and Institutional Review Board of the University of California, Irvine. The area chief, sub-chief and village elders were sensitized on the study activities planned, household heads provided written consent authorizing the spraying of their houses in the indoor residual spraying programmes. For mosquito collection, oral consent was obtained from the field owners in each location. These locations were not protected land, and the field studies did not involve endangered or protected species.

### WHO tube resistance bioassay

Mosquito larvae were collected from the seven study sites, and brought to the insectary of KEMRI and reared to adults. Upon pupation, they were transferred to cages and allowed to emerge as adults, and then provided with 10 % sucrose in cotton swabs. Three to five days old female adults were used for resistance bioassays. Female mosquitoes aged 2–5 days old were exposed to diagnostic doses of lambdacyhalothrin (0.05 %), deltamethrin (0.05 %), and permethrin (0.75 %). The bioassays were carried out using standard WHO testing protocol [38]. The number of surviving mosquitoes was recorded after the 24 h recovery period. A total of 200 mosquitoes per site, for each insecticide was tested.

### Species identification

Susceptible and resistant mosquitoes preserved after the bioassays were identified to species level using species-specific PCR assay following procedure described by Scott et al. [39]. At least 50 mosquitoes from every site preserved from the bioassays were used. A total of 1002 specimens within *An. gambiae* species complex were molecularly identified by PCR. DNA was extracted from the combined legs and wings of each specimen using ethanol precipitation [40]. The rDNA-PCR method was used to distinguish between the two sibling species of the *An. gambiae s.l.* species complex native to western Kenya, *An. gambiae s.s.* and *An. arabiensis* [40].

### Characterization of *kdr* mutation

PCR-based assay was carried out to check for *kdr* gene mutations in randomly selected individuals. A total of 570 mosquitoes were genotyped for the 7 populations. Extraction of DNA followed the procedure by Collins et al. [40]. Real-time TaqMan assay was used to quantify the genotype at amino acid position L1014S of the voltage gated sodium channel, following the methods of Bass et al. [41] as modified by Mathias et al. [31]. Samples were genotyped for the wild-type (susceptible) allele using probe 5´-CTTACGACTAAATTTC-3´, and for the 1014S *kdr* allele using probe 5´-ACGACTGAATTTC-3´.

### Evaluation of insecticidal decay effect of LLINs

Olyset® LLINs, the most commonly used LLINs in western Kenya, were collected from the field and used for insecticidal decay test. Olyset® is made of single filament polyethylene, blended with permethrin 2 % as active ingredient at a concentration of 1000 mg permethrin per m^2^ [42]. Twenty LLINs were collected randomly from two villages, and the household heads were surveyed for the age of the nets, and then provided with new LLINs. The net age was further checked by questionnaire survey to the spouse of the household heads on the months that started to use the LLIN, and by check with village chiefs and health center administrators on LLIN mass distribution years and months. The nets were packed individually in polythene bags, and then wrapped in aluminum foil and stored at 4 °C. Six pieces of netting materials, 30 × 30 cm^2^, were cut with two pieces each from the roof panel, upper side panel and lower side panel from each net [43]. Three pieces from each position were used for mosquito bioassays to determine their insecticidal effects, and the remaining three pieces for insecticide concentration analysis.

The insecticidal decay effect of LLINs was measured using the standard WHO cone bioassay [43]. Mosquitoes reared from field-collected larvae were used, and the susceptible Kisumu strain was used as a control. Ten female mosquitoes were released in each cone and exposed to the netting material for 3 min. They were then transferred to paper cups and held for 24 h. The number of mosquitoes knocked down was recorded 60 min after exposure, and the mortality rates scored after the 24 h recovery period. New unused LLINs were used as positive control. Each Net was tested with twelve replicates and 120 mosquitoes from each site.

### Net insecticide concentration analysis

To determine the insecticide concentration in the LLINs, 3 pieces of netting materials, 25 cm^2^ each, were cut from the roof panel, upper side panel and lower side panel, and placed in a glass test tube. The netting materials were immersed in the extraction solvent (4:1 hexane: chloroform solution) and then vortexed for 1 min. The netting materials were incubated at room temperature for 10 min, and then filtered through 0.45 μm PTFE (Polytetrafluoroethylene) membrane. The contents were diluted to a final volume of 10.0 ml. An aliquot of the 10.0 ml solution was analyzed by gas chromatography [44]. Molecular grade of permethrin and deltamethrin with known concentration (1 mg/ml in 4 hexane: 1 chloroform, stabilized with 0.1 % acetic acid) was used as internal standard to quantify the concentrating of permethrin and deltamethrin. Three independent insecticide extractions and gas chromatography analyses were conducted for each net.

### Insecticidal decay of insecticides in the IRS program

The IRS program in Kenya commonly uses lambda-cyhalothrin (ICON 10CS). 100 g of lambda-cyhalothrin (Trade name ICON 10cs 100 g/liter) was diluted in 1 L water, then 62.5 ml of the diluted insecticide was further diluted in 10 L water which was sprayed on the wall as recommended by the manufacturer. The insecticides were sprayed using Hudson X-pert compression sprayer (10 L capacity). ICON 10CS is designed by the manufacturer to be effective on the wall for up to six months [20]. To determine the insecticidal effects of insecticides used in IRS, five houses were randomly selected in the area where the IRS with ICON was on going. Mud slabs of 5 cm diameter were prepared with the same mud used for house walls by the local residents. Eight mud slabs in lid tops were attached to the wall in each selected house. Study houses were then sprayed using ICON 10CS capsule suspension. For each house, one mud slab was removed immediately after spraying, then each month for insecticidal activity bioassay in the subsequent seven months after spray.

The bioassays were conducted using the standard WHO cone bioassay with adult mosquitoes raised from field-collected larvae from the seven study sites. Twenty female *An. gambiae* mosquitoes were released into each cone at the vertical position. The mosquitoes were exposed to the treated surfaces of mud slab for 30 min. Mosquitoes were transferred to a recovery cup, and the number of surviving mosquitoes was recorded after the 24 h recovery period. Laboratory reared susceptible Kisumu strain, a WHO designated susceptible reference strain, was used as a control. For every month 5 replicates were used.

### Data analysis

The mortality rates of mosquitoes in the standard WHO tube resistance bioassay was calculated and adjusted using the Abbot’s formula [45]. Susceptibility status of a mosquito population was classified according to the WHO criteria (98–100 % mortality indicates susceptibility, 90–97 % mortality suggests possibility of resistance that needs to be confirmed, and <90 % mortality suggests resistance) [38]. LLINs were grouped into four classes: new net, <1 year, 1 ~ 2 years and >2 years. The mortality rates in the cone bioassays were calculated for each net age class for each of the two study sites. One-way analysis of variance (ANOVA) and Tukey-Kramer HSD test were used to determine the statistical difference between LLINs in different age groups. Finally, insecticidal decay effects of insecticides in the IRS were determined by comparing mortality rate in the cone bioassay among mud slabs at different months after spray. As in the LLIN insecticidal decay effect studies, one-way ANOVA and Tukey-Kramer HSD test were used to determine the statistical difference between mud slabs in different months after spray. Heterozygous and homozygous mutation rates of *kdr* gene were calculated. To determine if these genotypes were under selection, Hardy-Weinberg equilibrium test for *kdr* genotypes was performed, and *χ*^2^ test was used to determine the significance of the departure from Hardy-Weinberg equilibrium.

## Results

### Insecticide resistance status of the study populations

**Fig. 2:**
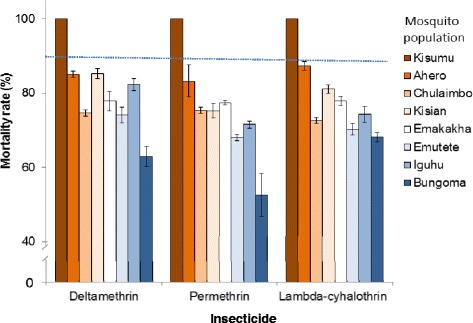
Mortality rates of *Anophlese gambiae* and *An. arabiensis* in the standard WHO tube insecticide susceptibility test. Standard diagnostic dosage was used for each insecticide: deltamethrin (0.05 %), permethrin (0.75 %) and lambda-cyhalothrin (0.05 %). The 95 % confidence interval is shown

WHO tube resistance bioassay found the Kisumu susceptible strain was highly susceptible to pyrethroids, but all 7 study populations were resistant as mortality rates were all below 90 % by WHO standards (Fig. 2) [38]. In particularly, 4 out 7 populations (Chulaimbo, Emakakha, Emutete and Bungoma) were highly resistant to deltamethrin as evidenced by <80 % mortality in the bioassay. Similarly, 6 out of 7 populations were highly resistant to permethrin, and 5 out of 7 populations highly resistant to lambda-cyhalothrin. Ahero population in rice-irrigation area was least resistant to the three insecticides tested.

### Species identification

PCR analysis showed that *An. gambiae* was the predominant species in Bungoma comprising 90.0 % specimens within *An. gambiae* species complex. *An. gambiae* remained the dominant species in the highland: Iguhu (88.0 %), Emutete (94.0 %) and Emakakha (93.4 %; Table 1). In the lowland *An. arabiensis* was dominant two sites: Kisian (64.4 %) and Ahero (89.3 %). Overall, 3.6 % of the samples tested were not successfully amplified.

**Table 1:** Species composition (%) at the seven study sites in western Kenya

Study site	N	*An. arabiensis*	*An. gambiae*	Not determined
Ahero	56	89.3	5.4	5.4
Kisian	225	64.4	32.9	2.4
Chulaimbo	100	24.0	72.0	4.0
Emutete	200	3.5	94.0	2.5
Emakakha	61	3.3	93.4	3.3
Iguhu	300	8.0	88.0	4.0
Bungoma	60	3.3	90.0	6.7

### *Kdr* genotyping

Table 2 summarizes *kdr* genotyping results. Overall, *kdr* genotyping failed in 2.8 % of the samples. Homozyzygous *kdr* mutations of L1014S ranged from 73 to 88 % in *An. gambiae* s.s. dominant populations, whereas L1014S mutations were relatively low (7–31 %) in *An. arabiensis* dominant populations. In addition, in *An. gambiae* most of the mutations were homozygous (Table 2). Hardy-Weinberg analysis found that, for the four *An. arabiensis* populations tested for Hardy-Weinberg equilibrium, only one population (Chulaimbo) showed significant deviation and the deviation resulted from heterozygosity deficiency (Table 2). On the other hand, for *An. gambiae*, five out of the six populations tested showed significant departure from Hardy-Weinberg equilibrium, and all were caused by heterozygosity deficiency.

**Table 2:** Genotype and allele frequencies of *kdr* at the seven study sites in western Kenya

Study site	*An. gambiae*						
	N	LL	LS	SS	Frequency	*χ* ^2^	*P*-value
Ahero	3	3	0	0	0.0	-	-
Kisian	50	32	3	15	33.0	37.35	<0.0001
Chulaimbo	56	2	4	50	92.9	11.93	<0.0001
Emutete	87	7	4	76	89.7	49.22	<0.0001
Emakakha	57	1	7	49	92.1	1.38	0.24
Iguhu	108	10	7	91	87.5	53.48	<0.0001
Bungoma	53	5	5	43	85.8	19.83	<0.0001
	*An. arabiensis*						
	N	LL	LS	SS	Frequency	*χ* ^2^	*P*-value
Ahero	50	46	4	0	4.0	0.09	0.77
Kisian	42	41	1	0	1.2	0.01	0.94
Chulaimbo	23	14	0	9	39.1	23.00	<0.0001
Emutete	0	N/A	N/A	N/A	N/A	-	-
Emakakha	1	1	0	0	0.0	-	-
Iguhu	16	15	1	0	3.1	0.02	0.90
Bungoma	3	2	0	1	33.3	-	-

N is sample size, LL represents wild type, LS represents heterozygote mutation, SS represents homozygote mutation, and Frequency is the mutation allele frequency (%). N/A means not applicable, and symbol ‘-‘stands for not done. *χ* ^2^ and P-value are the results of Hardy-Weinberg equilibrium test

### Insecticidal decay of LLINs with age

**Fig. 3:**
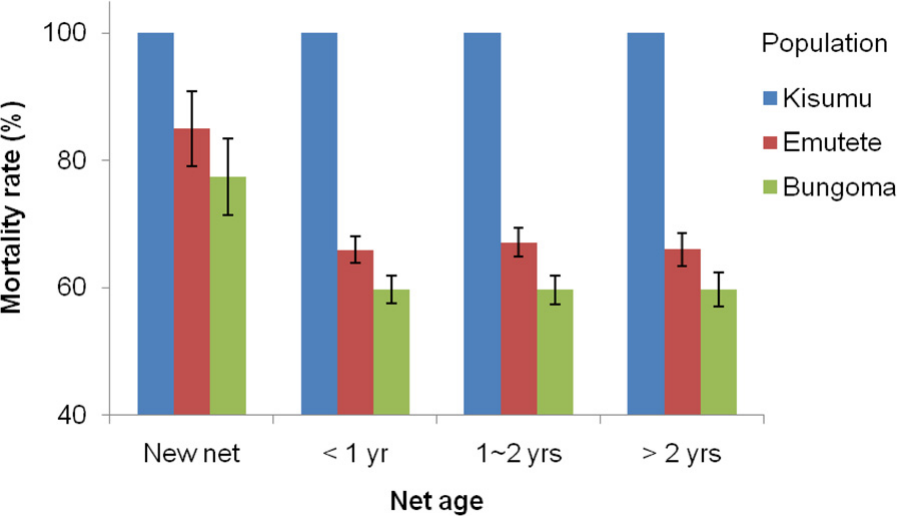
Mortality rates of field mosquito populations (Emutete and Bungoma) and laboratory susceptible Kisumu strain exposed to LLINs of various ages in the standard WHO cone bioassay. The 95 % confidence interval is shown

A total of 21 LLINs were tested using the WHO cone bioassay for their insecticidal activities. No surviving mosquitoes were observed in the cone bioassay, and there was no decrease in insecticidal activities in the LLINs for up to 3 years against the laboratory susceptible Kisumu strain (100 % mortality) (Fig. 3). However, mortality rate was decreased to 77.5 – 85.0 % when the new LLINs were tested against field collected mosquitoes, suggesting that the LLINs had limited killing efficacy on mosquitoes predisposed with insecticide resistance genes. The insecticidal activity in LLINs of 1 ~ 2 years and >2 years old was similar for both populations (66.7 % for Emutete and 59.6 % for Bungoma), but it was significantly lower than the new LLINs (85 % for Emutete and 77.5 % for Bungoma, *P* < 0.05) (Fig. 3).

**Fig. 4:**
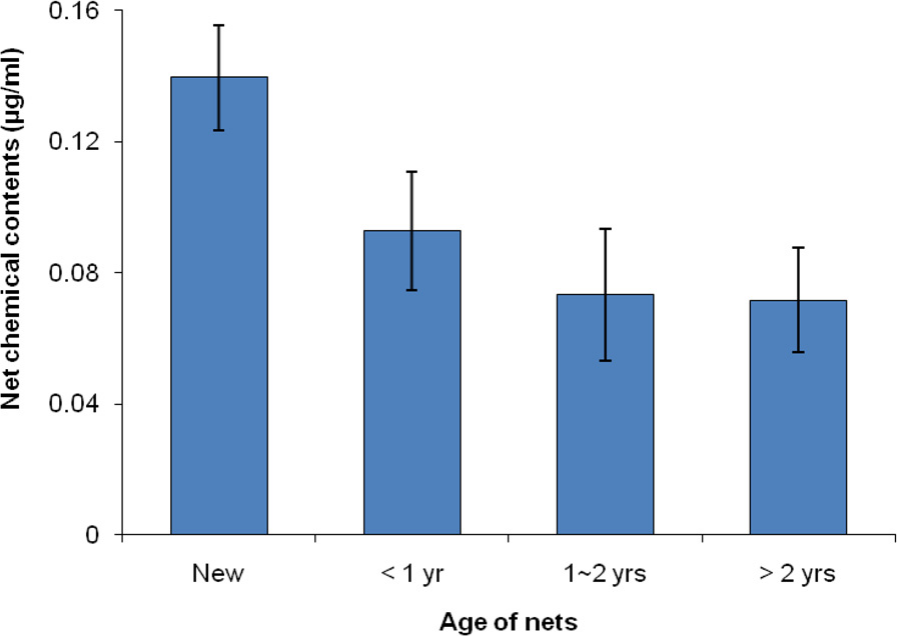
The net chemical content of the LLINs. The 95 % confidence interval is shown. The target concentration of permethrin for new LLINs was 0.14 % of total weight

The chemical concentration analysis found that new LLINs had the highest concentration at 0.14 μg/ml (Fig. 4). Significantly lower concentration (0.07 μg/ml) was found in LLINs of 1 ~ 2 years and >2 years old, in comparison to LLINs of <1 year old (0.09 μg/ml, *P* < 0.05), suggesting reduced availability of the active insecticide ingredient in the nets over time (Fig. 4).

### Insecticidal decay effect in mud walls sprayed with insecticides

**Fig. 5:**
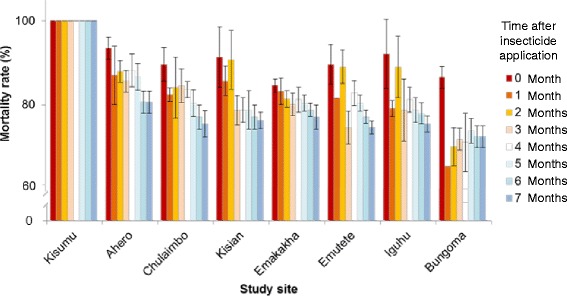
Bioassay mortality rates of seven mosquito populations and the susceptible Kisumu strain exposed to mud slabs treated with ICON (lambda-cyhalothrin). The 95 % confidence interval is shown

The mud slabs sprayed with lambda-cyhalothrin in the IRS program killed all susceptible *An. gambiae* for at least 7 months after the spray in the cone bioassay (Fig. 5). The mortality rate of freshly sprayed mud slabs ranged from 93.5 to 84.6 % for the 7 study populations, and such a reduction from 100 % mortality rate observed in the susceptible mosquito population likely resulted from insecticide resistance in the mosquito populations. Within the first four months after the spray, small reduction in the mortality rate was observed (71.1 – 88.2 %). Significant decay in insecticidal activities was observed 6 months after spray for most populations (72.5 -80.7 %, *P* < 0.05). Bungoma population, the most resistant population, consistently exhibited the lowest mortality rate during the test period (72.5 – 86.6 %), whereas the least resistant Ahero population showed the highest mortality rates during the testing periods (80.7 – 93.5 %).

## Discussion

Malaria transmission reduction by indoor residual spray and insecticide-impregnated nets is primarily through decreasing vector abundance and human-vector contact rate. Insecticidal efficacy of the nets and residual insecticides in IRS is critical to the efficacy of the malaria vector control program. Insecticidal efficacy may be affected by insecticide resistance in the mosquito vectors, gradual loss of chemical bioavailability and chemical degradation. This study was carried out to assess insecticidal efficacy of LLINs that have been used by residents for several years and to determine the efficacy of residual insecticides in the IRS program in the context of various insecticide resistance levels. Our data supports three conclusions: 1) Prior to the indication of decay, there was evidence that insecticide resistant mosquitoes were less likely to be killed after exposure to LLINS and IRS; 2) the LLINs we tested (Olyset®) showed time-dependent decay of insecticide concentration, and this was collaborated by reduced mortality in the older nets compared to new nets and by the insecticide chemical analysis in the nets; and 3) residual insecticide of IRS with lambda-cyhalothrin (ICON 10CS) showed decay in insecticidal efficacy 5 months after the spray in the field. Decay of pyrethroid insecticidal efficacy was indicated by bioassay mortality rates, instead of chemical assays as there were challenges in extracting pyrethroids from the sprayed surfaces.

*An. gambiae* from all study sites showed resistance to pyrethroids except *An. arabiensis* mosquito population in Ahero which was moderately susceptible to pyrethroids. The observed pyrethroid resistance in these regions could be linked to the public health use of insecticides for LLINs and IRS [31]. *An. arabiensis* in Ahero are largely exophilic and zoophilic [46], indicating its low contact with LLINs and sprayed walls and thus less exposure to the insecticide. Previous studies in these sites have reported high abundance of *An. gambiae* or complete lack of *An. arabiensis* in Bungoma, Emutete and Iguhu [33, 36, 47], the present study found 3–8 % of *An. arabiensis*, suggesting *An. arabiensis* was spreading and establishing the populations in western Kenya highlands. We found high frequencies of *kdr* mutations in *An. gambiae* and low frequencies in *An. arabiensis*, consistent with the finding of Ochomo et al. [33] which reported fixation of L1014S in Bungoma *An. gambiae* population and absence in *An. arabiensis* population. However, Kawada et al. [48] reported a high frequency and wide distribution of L1014S in *An. arabiensis* in Suba district, western Kenya, suggesting a patchy distribution pattern of *kdr* mutation in *An. arabiensis* in western Kenya.

The mortality rates of field mosquito populations against residual insecticide in IRS were significantly lower than the susceptible Kisumu strain, and decreased significantly with time. Lambda-cyhalothrin (ICON CS10) is known to persist on sprayed surfaces for six months [49]. Thus, reduced mortality against field mosquito populations in the freshly sprayed surface was resulted from insecticide resistance. On the other hand, insecticidal efficacy decay was evident five months after the spray. The magnitude of the effects of insecticide resistance and insecticidal decay were comparable, about 20 % less mortality (compare to control) in resistance and 15-20 % less mortality in insecticidal decay (compare to newly sprayed walls with after 6 months of spray). The lower mortality rates observed on treated mud wall could also be due to less bioavailability of the insecticides on the sprayed surfaces. Studies from different countries such as Tanzania and Vietnam also found that the material of walls affected the durability of insecticides sprayed on the wall [16, 50–53], a factor should be considered when choosing the insecticides for indoor residual spraying as many rural areas in tropical Africa are shifting from building mud walled houses to modern cemented houses. For example, studies in Tanzania with ICON 10 CS recorded 100 % mortality of *An. gambiae* up to seven months on sprayed mud wall surfaces [52], whereas in Vietnam, the residual effect of ICON lasted for up to four months on wood, five months on bamboo, and three months on bricks in bioassays against *An. dirus* [53]. A WHOPES supervised trial in Benin reported the residual effect of CS and WP ICON formulations with 30 mg/m^2^ to be up to two months only, and in India trials reported persistence up to four to six months [53]. The bioefficacy and persistency of insecticides, as revealed by mosquito mortality, depends on the type of surface, the dosage, and the age of spray deposits [50–55]. We speculate that microclimate conditions may also play a role.

Net cone bioassays and net chemical analysis confirmed the degradation of insecticides and its impact on mosquito mortality. Earlier studies have indicated the reduced efficacy of the nets with the number of washes, physical condition of the nets and insecticide resistance [24, 56]. Our finding is consistent with Toe et al. [57] who reported reduced susceptibility of field collected mosquitoes to LLINs, compared to laboratory reared Kisumu strain. Whether the observed insecticidal decay and insecticide resistance may lead to operational failure of LLINs is not clear. For example, whether this resistance could result in operational ITN or IRS malaria control failure in the field is unknown. For example, a report from Malawi found that the use of ITNs reduced the incidence of clinic malaria incidence by 30 % in children in an area with moderate levels of pyrethroid resistance and considerable malaria transmission [58]. A thorough assessment of the impact of this resistance on the efficacy of LLINs and IRS on malaria disease and transmission will give a clear indication to the decision makers on the need to shift or not from pyrethroids to alternative carbamate or organophosphate insecticides [59].

## Conclusion

In conclusion, this study used seven sites in malaria endemic and epidemic areas of western Kenya and confirmed that insecticide decay and developing levels of resistance have an important negative contribution to insecticidal efficacy of LLINs and IRS. This finding is congruent with several studies from this region over the past several years [3, 33, 48, 60–63]. Pyrethroid resistance poses a major threat to the current malaria control strategies in Africa. Due to increased residual malaria transmission and rebound of malaria transmission in many sites in Africa, transmission control is required despite of moderate and high insecticide pyrethroid resistance. In order to mitigate against the role of insecticide resistance and decay, new nets should be redistributed every three years as recommended by WHO while maintaining a high level of LLIN coverage and usage. There is also need for urgent development and deployment of non-pyrethroid based vector control tools and non-insecticide based ecological tools.
